# Effects of budlein A on human neutrophils and lymphocytes

**DOI:** 10.1590/1678-775720150540

**Published:** 2016

**Authors:** Carollinie Dias KNOB, Milena SILVA, Thaís Helena GASPAROTO, Carine Ervolino OLIVEIRA, Nádia Ghinelli AMÔR, Nilton Syogo ARAKAWA, Fernando Batista COSTA, Ana Paula CAMPANELLI

**Affiliations:** 1 - Universidade de São Paulo, Faculdade de Odontologia de Bauru, Departamento de Ciências Biológicas, Bauru, SP, Brasil.; 2 - Universidade Estadual de Londrina, Londrina, PR, Brasil.; 3 - Universidade de São Paulo, Faculdade de Ciências Farmacêuticas de Ribeirão Preto, Departamento de Farmacognosia, Ribeirão Preto, SP, Brasil.

**Keywords:** Lactones, Neutrophils, Lymphocytes

## Abstract

Sesquiterpene lactones (SLs) are the active constituents of a variety of medicinal plants used in traditional medicine for the treatment of inflammatory diseases and other ailments.

**Objective:**

In this study, we evaluated whether budlein A modulates the activation of innate and adaptive immune cells such as neutrophils and lymphocytes.

**Material and Methods:**

Our research group has investigated several plant species and several compounds have been isolated, identified, and their medical potential evaluated. Budlein A is a SL isolated from the species *Aldama buddlejiformis* and *A. robusta* (*Asteraceae*) and shows anti-inflammatory and anti-nociceptive activities. Advances in understanding how plant-derived substances modulate the activation of innate and adaptive immune cells have led to the development of new therapies for human diseases.

**Results:**

Budlein A inhibited MPO activity, IL-6, CXCL8, IL-10, and IL-12 production and induces neutrophil apoptosis. In contrast, budlein A inhibited lymphocyte proliferation and IL-2, IL-10, TGF-β, and IFN-γ production, but it did not lead to cell death.

**Conclusions:**

Collectively, our results indicate that budlein A shows distinct immunomodulatory effects on immune cells.

## INTRODUCTION

The ability of the immune system to respond to a variety of stimuli is central to its function in pathogen clearance and tissue repair^4^. The immune homeostasis and control of inflammation in different pathological settings is maintained by the balance between pro- and anti-inflammatory signaling^6^, and most research has focused on understanding the mechanisms that regulate the immune response^4^ ^,^ ^6^ ^,^ ^8^ ^,^ ^9^. New therapeutic agents are currently under investigation for pathological conditions in which the balance between activation and suppression of the immune response has failed^6^. Studies have shown that plant-derived compounds have immunomodulatory effects without direct immunosuppressive action such as clinically used corticosteroids^2^ ^,^ ^3^ ^,^ ^16^ ^,^ ^27^. However, the mere isolation of new naturally-occurring anti-inflammatory agents does not necessarily lead to a drug until the exact mechanism of action and their possible toxic effects are completely determined^9^. Moreover, many biologically active compounds that do not afford a new drug or even a principal compound can be used as prototypes for the study of the mechanisms involved in a certain biological effect. Budlein A, a sesquiterpene lactone (SL) formerly isolated from the Central American species *Aldama buddlejiformis* (DC)^1^ and more recently from the South American *A. robusta Gardner* (*Asteraceae*), has been described as presenting anti-inflammatory activity^1^ ^,^ ^7^ ^,^ ^27^. Recent studies have shown its anti-nociceptive and anti-inflammatory activities in mice^22^ ^,^ ^27^. Although this information is important for determining its properties, it provides restricted parameters about how this compound affects other cells, adaptive immune response and T cell compartment. An immunomodulatory substance might show antibacterial activity, but the induced cell death could also be harmful to immune responses, pathogen clearance and tissue repair^11^ ^,^ ^18^. In this context, research to develop new anti-inflammatory agents to control the damage and exacerbated inflammation mediated, in part, by lymphocyte activation, has been stimulated. Given the broad information relating SLs and immunomodulation, we should evaluate whether budlein A modulates the activation of innate and adaptive immune cells.

## MATERIAL AND METHODS

### Budlein A

Budlein A (MW=374.1365) was isolated from the leaves of *Viguiera robusta*, as previously described^27^. Its chemical structure was determined by means of spectrometric analysis, i.e., IR, 1H, and 13C nuclear magnetic resonance (NMR) spectrometry as well as comparison with an authentic sample and data reported in literature^5^. Analytical procedures using spectroscopy and chromatographic techniques indicated that the purity of budlein A used in this study ranged between 95-98%. Prior to the bioassays, budlein A was dissolved in dimethylsulfoxide (DMSO) (SIGMA – St. Louis, MO, USA) (0.1% in RPMI 1640 medium – GIBCO – Grand Island, NY, USA).

### Healthy volunteers

We used peripheral blood from 30 healthy volunteers (17 men and 13 women; age ranged between 27-50 years). All subjects had no active diseases at the time of phlebotomy. All subjects signed an informed consent releasing the use of blood for research purposes approved by the Bauru School of Dentistry, University of São Paulo.

### Isolation of leukocytes

Peripheral blood was harvested with heparin (50 U/ml) from healthy subjects. Both groups of leukocytes were layered over two different density Histopaque gradients (SIGMA) and centrifuged for 30 min at 450X g. Peripheral blood mononuclear cells (PBMC) were obtained from the first layer (onto Histopaque 1077) and neutrophils were isolated from the second buffy coat layer (onto Histopaque 1119). Cells were then washed twice with Roswell Park Memorial Institute medium (RPMI) at 200 X g for 10 min before being quantified. Viability of the cells was >99% viable when assessed by Trypan blue exclusion.

### Neutrophils culture

Neutrophils (1x10^6^/well) from healthy donors were cultured in 24-well plates, in presence of medium only, or Lipopolysaccharide (LPS) (10 ng/mL), N-formyl-methionine-leucine-phenylalanine (fMLP) (1.5 µM) in presence or absence of different concentrations of budlein A (1, 10 or 100 µM) or dexamethasone (100 µM).

### T cell culture and proliferation assays

Carboxyfluorescein Succinimidyl Ester (CFSE) (Invitrogen – Eugene, OR, USA) labeled-PBMC (1x10^6^/well) from healthy donors were cultured in 24-well plates, in the presence of phytohaemagglutinin (PHA) (1 µg/ml) (SIGMA), or in presence or absence of different concentrations of budlein A (1, 10, or 100 µM) or dexamethasone (100 µM), at 37°C and 5% CO_2_. On day 4, the cells were harvested and the proliferative response of T cells was assessed by measuring the CFSE dilution using flow cytometry. The proliferation of T cells was characterized by sequential halving of CFSE fluorescence, generating equally spaced peaks on a logarithmic scale. The data represents the percentage of proliferation of T cells. The cell acquisition was performed on a FACSort flow cytometer using CellQuest software (BD Biosciencess – San Diego, CA, USA).

### Myeloperoxidase activity

Myeloperoxidase (MPO) activity was determined by enzymatic reaction, as previously described^26^. Neutrophils were harvested after culture and centrifuged at 350X g for 15 min, and the pellet was frozen at -20°C. The pellet was then liquefied and centrifuged twice at 10,000X g for 15 min at 4°C. The MPO activity in the suspended pellet was assayed by measuring the change in absorbance at 450 nm using tetramethylbenzidine (1.6 mM) and H_2_O_2_ (0.5 mM) (BD Biosciences).

### Cytokine assays

Interleukin (IL)-2, IL-6, CXCL8, IL-10, IL-12, Transforming Growth Factor (TGF)-β, and Interferon (IFN)-γ levels were measured using ELISA kits (BD Biosciences or R&D Systems – Minneapolis, MN, USA), according to the manufacturer’s instructions.

### Apoptosis

Cells were harvested after culture and the viability was analyzed by flow cytometry, as previously described^26^. The percentage of apoptotic cells was calculated from the proportion of neutrophils, as well as lymphocyte positivity for Annexin- V-FITC or propidium iodide (ApoScreen™ Annexin V-FITC kit, SouthernBiotech – Birmingham, AL, USA), in relation to total number of cells^26^.

### Statistical analysis

Values are presented as mean±SEM. Statistical analysis was performed using ANOVA followed by the Tukey’s multiple comparison test (INSTAT Software; GraphPad Prism, La Jolla, CA, USA). All values were considered significant when p≤0.05.

## RESULTS

### Budlein A inhibits MPO activity, cytokine production, and induces neutrophil apoptosis

**Figure 1:**
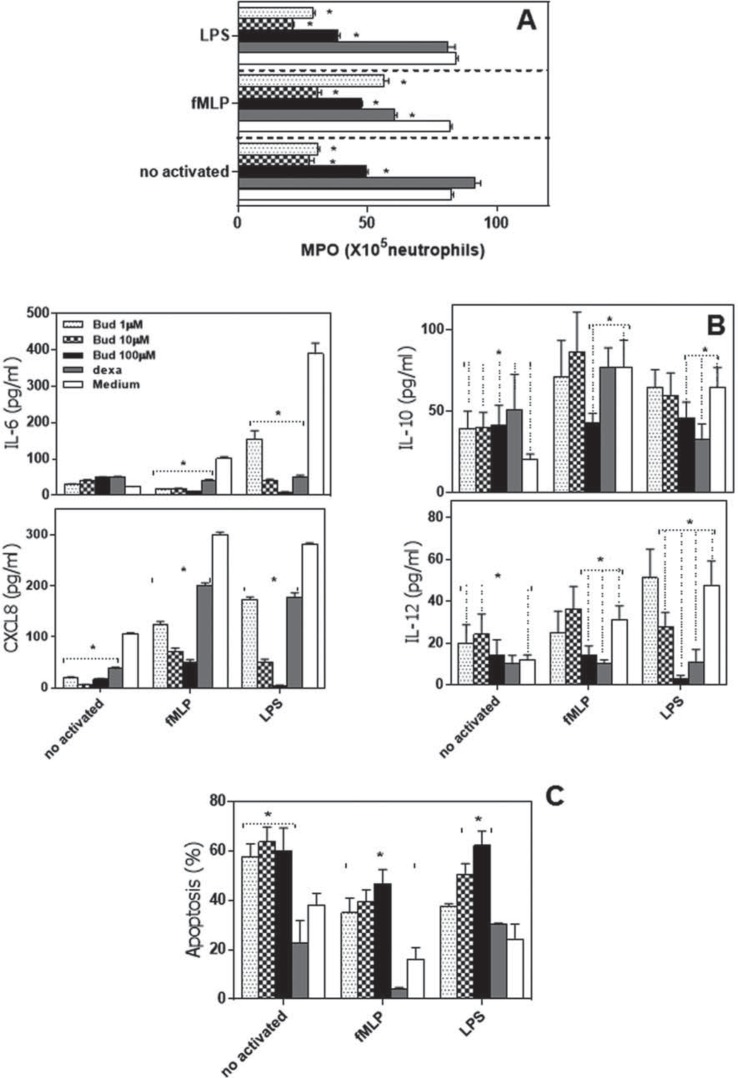
Budlein A negatively regulated the neutrophil activation to fMLP and LPS. Neutrophils from healthy controls were cultured with medium only or budlein A (1 μM, 10 μM, or 100 μM), dexamethasone (100μM), budlein plus fMLP, or budlein plus LPS. (A) Analysis of myeloperoxidase activity (MPO). B) IL-6, CXCL8, IL-10, and IL-12 production were analyzed by ELISA. (C) Flow cytometric analysis of apoptosis. The bars represent the mean±SEM for each volunteer individually tested. * p<0.05

To evaluate the anti-inflammatory effects of budlein A on neutrophils, cells were stimulated with fMLP or LPS in presence or absence of budlein A (1, 10, or 100 µM) or dexamethasone (positive control), and the myeloperoxidase (MPO) activity, IL-6, CXCL8, IL-10, and IL-12 production and apoptosis were analyzed. Consistent with prior observation^27^, budlein A inhibited in a concentration-dependent manner the MPO activity by LPS-stimulated-neutrophils (Fig. 1A). Our results also indicate that at a concentration of 10 and 100µM, budlein A reduced MPO activity by fMLP-stimulated neutrophils (Figure 1A).

Because neutrophils appear to be important producers of cytokines during innate immune response and cytokines are important mediators of the inflammatory process, we assessed whether budlein A affects cytokine and chemokine production by neutrophils (Figure 1B). Unstimulated neutrophils produced basal levels of IL-6, IL-10, CXCL8, and IL-12. Budlein A decreased CXCL8 production, induced IL-10 production and did not alter IL-12 and IL-6 production by unstimulated neutrophils. After 21 h of culture, LPS and fMLP enhanced IL-6, CXCL8, IL-12, and IL-10 production by human neutrophils. In contrast, budlein A inhibited in a concentration-dependent manner IL-6 and CXCL8 production by fMLP and LPS-stimulated neutrophils. Moreover, budlein A, at only 100 µM concentration, inhibited IL-10 and IL-12 production by fMLP and LPS-stimulated-neutrophils, while at 10 µM it inhibited IL-12 production by LPS-activated neutrophils (Figure 1B). To verify whether the results were the consequence of cell death, we analyzed the apoptosis of neutrophils. Our data showed that only budlein A at 100 µM concentration significantly induced neutrophil apoptosis (33.19±5.70%), similar to that observed after culture with dexamethasone (22.83±0.51%) (Figure 1C). Similar results were observed when fMLP or LPS-stimulated neutrophils were treated with budlein A (100 µM) (Figure 1C).

### Budlein A inhibited T cell proliferation

**Figure 2:**
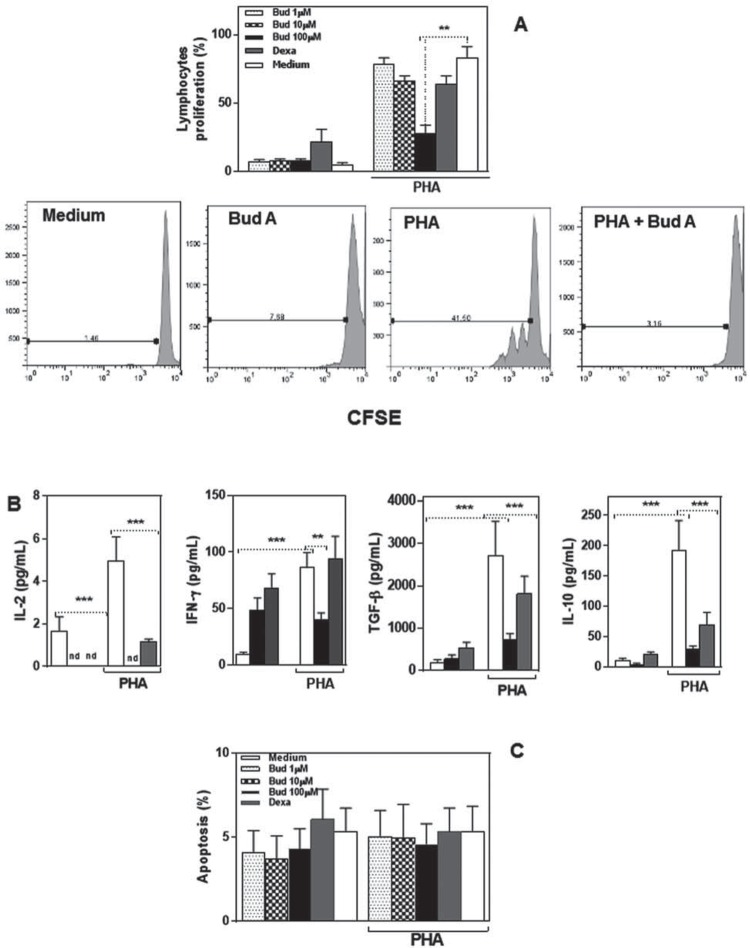
Budlein A inhibited T cell proliferation. (A) Flow cytometric analysis of proliferative response of PBMC cultured with Budlein A, dexamethasone, and PHA (1.5 mM) for 96 h. (B) IL-2, IFN-gamma, TGF-beta, and IL10 concentration in supernatant were analyzed by ELISA. (C) Flow cytometric analysis of apoptosis. The bars represent the mean±SEM of values obtained for each subject individually tested. *p<0.05, **p<0.01, ***p<0.001 was considered statistically significant when compared with the medium

To determine the anti-inflammatory effects of budlein A on lymphocytes, cells were cultured with PHA in presence or absence of budlein A (1, 10, or 100 µM) or dexamethasone (positive control), and proliferation, IL-2, IL-10, IFN-γ, and TGF-β production and apoptosis were analyzed. Lymphocytes exhibit proliferative response after PHA stimulation, and the addition of budlein A (100 µM) significantly reduced T cell proliferation (Figure 2A). These results indicate that budlein A interferes with PHA-induced T cell proliferation. Regarding cytokine production, the results evidenced that budlein A (100 µM) decreased IFN-γ, IL-2, IL-10, and TGF-β production by PHA-stimulated lymphocytes, with similar effects to dexamethasone (Figure 2B).

Since budlein A negatively modulates the secretion of cytokines by PHA-stimulated lymphocytes, and this could be a consequence of cell death, their apoptosis was evaluated (Figure 2C). We found that budlein A (1, 10, or 100 µM) does not induce lymphocyte apoptosis (Figure 2C). These data indicate that budlein A exerts an important role against lymphocytes’ activation, but it did not induce cell death.

## DISCUSSION

Although budlein A has been described as presenting anti-inflammatory and anti-nociceptive activity^1^ ^,^ ^7^ ^,^ ^27^, specific effects of this compound on immune cells are lacking. In the present study, we demonstrated that budlein A inhibit neutrophils activation and induce cell death, but, despite also inhibiting lymphocyte activation, it did not induce lymphocyte death.

Budlein A inhibited in a concentration-dependent manner the release of biochemical mediators by neutrophils, and this activity could possibly reduce tissue damage caused by the enzyme^24^ ^,^ ^28^. The MPO activity can be deleterious to the host, and it has been implicated in the pathogenesis of many inflammatory diseases^21^. Myeloperoxidase has also been established as a risk factor for many forms of cancer via its ability to damage/mutate cellular DNA or indirectly via its pro-inﬂammatory role^9^ ^,^ ^21^. The anti-inflammatory activity of SL is mediated via the inhibition of nuclear factor-kappa B (NF-κB) by the alkylation of the cysteine residue in the p65 subunit^18^, impairing its activation and the consequent production of inflammatory mediators^15^ ^,^ ^17^.

Another important effect of budlein A on neutrophils shown in this study was the inhibition of IL-6, CXCL-8, IL-10, and IL-12 production. Pro-inflammatory cytokines (IL-6, CXCL8, and IL-12) are involved in the initiation and amplification of the immune response, and anti-inflammatory cytokines modulate the late events to control the immune response^23^. The imbalance between pro- and anti-inflammatory cytokine activities favors chronic inflammation and autoimmune diseases^8^ ^,^ ^20^ ^,^ ^23^. Imbalance in the production of inflammatory cytokines may allow a local inflammation to become an uncontrolled systemic inflammatory response^8^ ^,^ ^20^ ^,^ ^21^.

Because low cytokine production might have occurred as a consequence of cytotoxic effects exerted by budlein A, we analyzed the apoptosis of human neutrophils. Only budlein A, at the 100 µM concentration, induces neutrophils apoptosis. The death of neutrophils observed in this study may be due to the absence of growth factors, particularly CXCL8, since budlein A, as well as other sesquiterpenes, inhibits activation of NF-κB and the production of cytokines related to neutrophil survival^1^ ^,^ ^23^. In fact, 1 µM of budlein A did not significantly induce neutrophil apoptosis, but it did not significantly inhibit the production of CXCL8 either. Another possible explanation could be a direct influence of the substance in neutrophil survival. Death of neutrophil induced by budlein A could be harmful to immune response, pathogen clearance, and tissue repair^24^ ^,^ ^25^. Neutrophils are involved with different inflammatory diseases and their apoptosis might generate an anti-inflammatory microenvironment as well^26^. The precise regulation of neutrophil apoptosis is essential for resolution of inflammation, since this prevents the release of toxic intracellular components that can damage healthy tissues^10^ ^,^ ^12^ ^,^ ^19^ ^,^ ^26^. Thus, anti-inflammatory agents that induced neutrophil apoptosis would only be helpful in some chronic inflammatory diseases, since these diseases present persistent inflammatory mediators avoiding their resolution^12^ ^,^ ^26^ ^,^ ^27^. A plethora of those inflammatory mediators (i.e., the reactive oxygen species or neutrophil extracellular traps) is produced by viable neutrophils^13^ ^,^ ^28^. These mediators could influence autoimmune diseases at different levels and by different ways, such as directly through the inhibition of cytokines, modulating their production or damaging surrounding tissues^14^ ^,^ ^28^. Several diseases seem to be affected by activated neutrophils, including rheumatoid arthritis (RA) and systemic lupus erythematosus (SLE)^13^ ^,^ ^14^ ^,^ ^28^. Hence, reduced numbers of activated neutrophils might generate a protected microenvironment from dramatic consequences from their uncontrolled inflammation^29^.

Interestingly, despite previous studies having shown that high concentrations of sesquiterpene lactones induced apoptosis of PBMC^30^, we did not find any significant change in lymphocyte death using three different concentrations of budlein A. Budlein A at 100 µM significantly inhibited PHA-proliferation of lymphocytes as well as IFN-γ, IL-2, IL-10, and TGF-β production. Other studies have described a significant inhibition of lymphocyte proliferation via other sesquiterpenes; however, the viability rate of the cells had not been evaluated^23^. Here, inhibition of lymphocyte proliferation might be a consequence of undetectable levels of IL-2, an important cytokine inducing lymphocyte proliferation. Thus, budlein A might be an important inhibitor of uncontrolled lymphocyte proliferation without inducing their apoptosis. Such situation would facilitate their use in diseases without directly affecting their survival.

**Figure 3:**
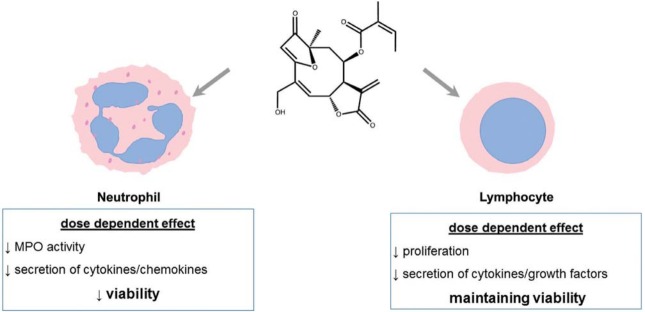
Graphical abstract showing effects of Budlein A on human neutrophils and lymphocytes

Therefore, our results also show that budlein A induced apoptosis of neutrophils but not lymphocytes, and indicate that budlein A shows distinct immunomodulatory effects on immune cells (Figure 3).

## CONCLUSIONS

The anti-inflammatory activities mediated by budlein A reveal an important potential to the study of mechanisms of action and therapeutics^1^ ^,^ ^5^. In the present study, we demonstrated that budlein A inhibits neutrophil activation and induces cell death, but, despite also inhibiting lymphocyte activation, it did not induce lymphocyte death. Further studies are necessary to understand the significance of this compound in the treatment of chronic inflammatory diseases. It will be of interest to evaluate how the balance between these positive and negative effects could be regulated during the course of the immune response.
